# Growth and endocrinopathies among children with β-Thalassemia major treated at Dubai Thalassemia centre

**DOI:** 10.1186/s12887-024-04670-w

**Published:** 2024-04-05

**Authors:** Rabah Almahmoud, Amal Hussein, Fatheya Al Khaja, Ahmed Farrag Soliman, Hany Dewedar, Zainab Al Shareef, Sarah Mathai

**Affiliations:** 1 Department of Clinical Sciences, College of Medicine, University of Sharjah, Sharjah, United Arab Emirates; 2 Department of Family & Community Medicine and Behavioral Sciences, College of Medicine, University of Sharjah, Sharjah, United Arab Emirates; 3 Dubai Thalassemia Centre, Dubai Health Authority, Dubai, United Arab Emirates; 4 Department of Basic Sciences, College of Medicine, University of Sharjah, Sharjah, United Arab Emirates; 5 Department of Pediatrics, Christian Medical College, Vellore, India

**Keywords:** Thalassemia, Endocrinopathies, Deferasirox, Hypothryroidism, Diabetes mellitus, Growth delay

## Abstract

**Background:**

β-Thalassemia major (BTM) is one of the most common hereditary anemias worldwide. Patients suffer from iron overload that results from repeated blood transfusion This in turn leads to multiple organ damage and endocrinopathies. This study aims to assess the prevalence of growth retardation, hypothyroidism, and diabetes mellitus in children and adolescents with BTM treated at Dubai Thalassemia Centre.

**Methods:**

A total of 105 children and adolescents were included in this retrospective observational study.

**Results:**

39 children and 66 adolescents’ data were analyzed. Females composed 51.3% (*n* = 20) of children and 53.0% (*n* = 35) of adolescents. Pretransfusion hemoglobin below 9 gm/dl was observed in 10.8% (*n* = 4) and 10.6% (*n* = 7) in children and adolescents, respectively. The mean age of menarche was 13.5 years. Among all study participants, 22.6% (*n* = 14) had normal height velocity whereas 37.1% (*n* = 23) had reduced height velocity in one year and 40.3% (*n* = 25) had reduced height velocity in two consecutive years. The proportion of children and adolescents showing reduced height velocity was significantly higher in females compared to the males (90.6% versus 63.3%, respectively, Chi-square = 6.597, p-value = 0.010). Although none of the study participants had diabetes mellitus, 26.1% (*n* = 12/46) had pre-diabetes. Elevated TSH was observed in 14.7% (*n* = 5) children and 8.1% (*n* = 5) adolescents while low FT4 was reported in one child and one adolescent.

**Conclusion:**

Of all endocrinopathies seen among children and adolescents with BTM, growth delay remains the main concern for this group of patients. Effective treatment is key to further reducing endocrinopathies. Although the sample size is limited, we postulate that the low percentage of endocrinopathies among children with BTM treated at Dubai thalassemia center and the low level of pretransfusion anemia reflect the effective transfusion and chelation at the center.

## Introduction

β-Thalassemia major (BTM) is a heterogenous group of hemolytic anemias that have an autosomal recessive inheritance. Anemia can result from reduced synthesis (β+) or complete absence (β0) of production of the β-globin’s hemoglobin chain [1, 2]. The complications of iron overload, arising from transfusions that represent the basis of disease management in most patients with severe thalassemia, might further complicate the clinical phenotype. Patients with BTM receive blood transfusion on regular basis loading the body with iron at a rate of 0.3-0.6 mg/kg/day and with the fact that the human body has poor mechanism of eliminating iron from the body [3]. The excess iron is deposited in different organs predominantly in the heart, liver, and endocrine glands giving rise to medical complications from iron toxicity, oxidative stress, and cell death [4]. The cardiac complications, such as left ventricular systolic and diastolic dysfunction, pulmonary hypertension, valvulopathies, arrhythmias and pericarditis, are the leading cause of death in patients with BTM [5]. Iron overload can disrupt the normal coaptation of the valve leaflets, resulting in incomplete closure and regurgitation of blood. Valvar regurgitation can lead to volume overload of the affected chambers of the heart, potentially causing further complications and impairing cardiac function [6]. Also, the liver is affected as iron overload can lead to liver failure and cirrhosis [7].

The endocrine system seems particularly sensitive to iron deposition. Data suggest that severe pituitary iron deposition occurs as early as the first decade in life and then leads to multiple endocrine complications at the level of hypothalamic- pituitary level [8]. Direct damage in the endocrine glands namely the thyroid, parathyroid, pancreas, and gonads occurs as well. Firstly, the thyroid gland is susceptible to cell death from the effect of free unbound iron particles, in addition to the effect of hypoxia from chronic anemia [9]. Secondly, the parathyroid gland, like any other gland, seems to be affected by toxic iron leading to subclinical hypoparathyroidism and less commonly to overt or symptomatic hypoparathyroidism with symptoms of hypocalcemia i.e. seizure and tetany [10]. Thirdly, data suggest that the pancreas is no exception to the toxic effect of iron. Studies have shown that adolescents treated with regular blood transfusion show evidence of insulin resistance and higher insulin secretion compared with non-thalassemia controls before the development of diabetes [11]. Furthermore, children and adolescents with poor or no chelation therapy showed more evidence of insulin resistance than their counterparts on iron chelation therapy [12]. Growth failure and hypogonadism are regarded among the commonest endocrinopathies in surviving thalassemia patients mainly due to hemosiderosis [13].

Dubai Thalassemia Centre is located in Dubai; one of seven Emirates constituting the United Arab Emirates; and except for the Emirate of Abu Dhabi, the Centre serves patients from all the other Emirates.

This study aims to assess the prevalence of endocrinopathies namely diabetes mellitus, hypothyroidism, growth delay, and delayed puberty among children and adolescents treated at Dubai Thalassemia Centre.

## Materials and methods

### Study design

This study has an observational retrospective cohort research design. Retrospective data, of all patients with BTM aged above two years and attended the Dubai Thalassemia Centre during the period of November 2019 and May 2021, were extracted from patients’ electronic medical records.

### Collection of data

In this retrospective study, data were extracted from the medical records as related to patients’ sociodemographic and clinical characteristics. Two separate data collection sheets were used to extract data from the records of male and female patients. For both genders, sociodemographic and physiological characteristics including date of birth, gender, nationality, patient’s weight and height were collected. The daily intake of dairy products, supplements and drug compliance were also collected. Data related to the secondary sex characteristics of females and males were recorded. Extracted clinical data included full blood count, thyroid profile, bone health, and liver function tests. The diagnosis of BTM was initially done based on the clinical presentation and was then confirmed by DNA analysis and hemoglobin electrophoresis.

### Data analysis

Data were initially entered on Microsoft Excel and were then exported to Statistical Package for Social Sciences (SPSS) program, version 28.0, for data coding and analysis. Descriptive data analysis was conducted using frequency distribution statistics (counts and percentages) for qualitative variables and measures of central tendency (means and medians) and variability (standard deviation and interquartile range) for quantitative data. Statistical tests were conducted as appropriate to the type of analyzed data. Means and standard deviations (SD) were reported for normally distributed data while medians and interquartile range (Q1-Q3) were reported for skewed data. To test the normality of quantitative data, Kolmogorov-Smirnov and Shapiro-Wilk tests were used while testing the equality of variances was performed using the Levene’s test. The independent t-test or Mann-Whitney U test were used to test the equality of two means or medians, respectively. One-way analysis of variance (ANOVA) or Kruskal-Wallis tests were used to test the equality of more than two means or medians, respectively. Missing data was handled using the pairwise deletion approach. A p-value below 0.05 indicated statistical significance.

Based on age, the cohort was classified as children (2–9 years) and adolescents(10–18 years). To measure the prevalence of diabetes mellitus, fasting blood glucose (FBG) level was categorized into three groups: normal (FBG < 100 mg/dL), prediabetes (FBG 100 mg/dL − 125 mg/dL) and diabetes (FBG ≥ 126 mg/dL) [14, 15]. Blood transfusion was performed to maintain the pretransfusion hemoglobin level above 9 g/dL as per thalassemia transfusion guidelines [16–18]. Thyroid-Stimulating Hormone (TSH) was dichotomized into two groups: normal (TSH between 0.3 and 4.2 ulU/ml) and elevated (TSH > 4.2ulU/ml). Free thyroxine (FT4) was also grouped into normal (FT4 12–23 pmol/L) and low (FT4 < 12 pmol/L). Elevated TSH along with normal Free T4 level was classified as primary hypothyroidism, while elevated TSH level with normal Free T4 level was classified as subclinical hypothyroidism. Growth delay was determined by annual height velocity less than the expected for age as follows: 2–4 years : < 7 cm/year, 4–6 years : < 6 cm/year, 6 years-till pubertal onset : < 4.5 cm/ year [19]. In addition, the height of each study participant was standardized by calculating its relative Z score, and consequently, patients with z score values below − 2 were considered to have short stature.

### Ethical approval

Data collection for this research commenced after granting ethical approval from Dubai Scientific Research Ethics Committee at Dubai Health Authority. The reference number for the approval letter is DSREC-06/2019-10.

## Results

This study analyzed data of a total of 105 children (*n* = 39) and adolescents (*n* = 66) with β thalassemia. The mean age was 5.38 years (sd = 2.145) for children and 15.22 years (sd = 2.361) for adolescents. Females composed 51.3% (*n* = 20) of children and 53.0% (*n* = 35) of the adolescents. Of all study participants, 45.5% (*n* = 46) were UAE nationals, 24.8% (*n* = 25) were Arabs, 29.7% (*n* = 30) were non-Arabs, and 3.8% (*n* = 4) had other nationalities. For adolescent girls, the mean age of menarche was 13.5 years (sd = 1.503). Among all study participants, 22.6% (*n* = 14) had normal height velocity whereas 37.1% (*n* = 23) had reduced height velocity in one year and 40.3% (*n* = 25) had reduced height velocity in two consecutive years. In other words, 77.4% (*n* = 48) of all children and adolescents had reduced height velocity at least in one year (Table 1). Among all study participants, short stature (Z score < -2) was reported in three children, two boys and a girl.

**Table 1:** Demographic and physical characteristics of children (2–9 years) and adolescents (10–18 years) with beta thalassemia

	Children (*N* = 39)		Adolescents (*N* = 66 )		Total (*N* = 105)	
	*n*	%	*n*	%	*n*	%
Age (years) ; Mean (sd)	5.38 (2.145)		15.22 (2.361)		11.57 (5.290)	
Gender						
Male	19	48.7	31	47.0	50	47.6
Female	20	51.3	35	53.0	55	52.4
Nationality						
UAE	16	42.1	30	47.6	46	45.5
Arabs	10	26.3	15	23.8	25	24.8
Non- Arabs	12	31.6	18	28.6	30	29.7
Diagnosis						
BTM	39	100.0	66	100.0	105	100.0
Height Velocity (2019-20)						
Normal	17	51.5	12	30.0	29	39.7
Reduced	16	48.5	28	70.0	44	60.3
Height Velocity (2020-21)						
Normal	13	52.0	13	35.1	26	41.9
Reduced	12	48.0	24	64.9	36	58.1
Height Velocity *(2 consecutive years)*						
Normal	6	24.0	8	21.6	14	22.6
Delayed in one year	15	60.0	8	21.6	23	37.1
Delayed in two years	4	16.0	21	56.8	25	40.3

The proportion of patients with pretransfusion hemoglobin below 9 gm/dl was 10.8% (*n* = 4) among children and 10.6% (*n* = 7) among adolescents. The median ferritin level in children was 2213 (IQR: 1636–3181) and 2521 (IQR: 1621–3789) in adolescents. Although none of the study participants had diabetes mellitus, 26.1% (*n* = 12 out of 46) had pre-diabetes. Elevated TSH was observed in 14.7% (*n* = 5) of the children and 8.1% (*n* = 5) of the adolescents while abnormal FT4 (FT4 < 12 pmol/L) was reported in only one child and one adolescent (Table 2). Children with elevated TSH levels had a median ferritin value of 2468 (IQR: 1596–3148) and were all within the normal range of FT4 (between 12 and 23). Blood transfusion was done by all the children and adolescents, with the majority performing blood transfusion once every three weeks. Regarding the chelation drug therapy, the majority of patients were on a single chelation drug (95.3%, *n* = 100) while only 3.8% (*n* = 4) used two drugs simultaneously. These four patients were adolescents while all children were on a single chelator. Of the total 105 study participants, one patient did not use chelation therapy as she had undergone bone marrow transplant. 14.3% (*n* = 15) used DFO, 2.9% (*n* = 3) used DFP, 78.1 (*n* = 82) were on DFX, 1.9% (*n* = 2) were on DFO and DFX, and 1.9% (*n* = 2) were on DFP and DFX. Among children, the drugs used for chelation therapy were Deferoxamine (DFO) (28.2%) and Deferasirox (DFX) (66.7%) while among the adolescents, DFO was used by 6.1%, Deferiprone (DFP) by 3.0%, and DFX by 84.8% (Table 2).

**Table 2:** Clinical characteristics of children (2–9 years) and adolescents (10–18 years) with beta thalassemia

	Children (*N* = 39)		Adolescents (*N* = 71 )		Total (*N* = 110)	
	*n*	%	*n*	%	*n*	%
Diagnosis						
BTM	39	100.0	66	100.0	105	100.0
Hemoglobin ; Mean (sd)	9.69 (0.829)		9.90 (0.986)		9.83 (0.934)	
Hemoglobin group						
< 9 gm%	4	10.8	7	10.6	11	10.7
≥ 9 gm%	33	89.2	59	89.4	92	89.3
Ferritin ; Median (Q1-Q3)	2213 (1636–3181)		2521 (1621–3789)		2361 (1644–3725)	
Fasting Glucose						
< 100 mg/dL	-	-	34	73.9	34	73.9
100 - < 126 mg/dL	-	-	12	26.1	12	26.1
≥ 126 mg/dL	-	-	0	0.0	0	0.0
TSH (uIU/ml)						
0.3–4.2	29	85.3	57	91.9	86	89.6
> 4.2	5	14.7	5	8.1	10	10.4
FT4						
< 12.0	1	2.9	1	1.5	2	2.0
12–23	33	97.1	64	98.5	97	98.0
Transfusion - Time of Initiation						
Days; Median (Q1 – Q3)	244 (182–455)		273 (120–1119)		260 (136–929)	
Years; Median (Q1 – Q3)	0.67 (0.50–1.24)		0.71 (0.31–3.03)		0.68 (0.35–2.54)	
Number of Transfusions per year						
Q3-4 W	5	27.8	6	9.8	11	13.9
Q3W	13	72.2	51	83.6	64	81.0
Q4W	-	-	3	4.9	3	3.8
Q6W	-	-	1	1.6	1	1.3
Drug of Chelation						
DFO (Deferoxamine)	11	28.2	6*	9.1	17	16.2
DFP (Deferiprone)	1	2.6	4*	6.1	5	4.8
DFX (Deferasirox)	26	66.7	60*	90.9	86	81.9

*Four adolescents were on two chelators; 2 were on DFO + DFX and 2 were on DFP + DFX

Ferritin level were done every 3 months and ferritin level was studied in association with demographic, physical and clinical parameters. Ferritin level significantly differed by the use of DFO chelation drug where ferritin level was 3034 among the DFO drug users compared to 2183 among the non-users (U = 1045, p-value = 0.004) (Table 3).

**Table 3:** Comparing Ferritin level by other parameters

	*n*	Ferritin level	Test value	*p*-value
Age group				
2–9	37	2212	1325*	0.394
10–18	65	2521		
Gender				
Male	50	2459	1328*	0.851
Female	52	2299		
Nationality				
UAE	45	2328	1.699^	0.428
Arabs	23	2722		
Non- Arabs	30	2299		
Height Velocity (2020 − 2019)				
Normal	28	2303	647*	0.596
Reduced	43	2468		
Height Velocity (2021 − 2020)				
Normal	26	2255	539*	0.221
Reduced	35	2600		
Height Velocity *(2 consecutive years)*				
Normal	14	2303	3.217^	0.200
Reduced in one year	23	2269		
Reduced in two years	24	2727		
DFO (Deferoxamine) Chelation Drug				
No	85	2183	1045*	***0.004***
Yes	17	3034		
DFP (Deferiprone) Chelation Drug				
No	97	2357	**	
Yes	5	4285		
DFX (Deferasirox) Chelation Drug				
No	19	2969	274*	0.625
Yes	83	2268		

*Mann-Whitney U test for comparison of two medians

^Kruskal Wallis test for comparing more than two medians

**Not calculated due to small number of cases

The proportion of children and adolescents showing reduced height velocity was significantly higher in females compared to the males (90.6% versus 63.3%, respectively, Chi-square = 6.597, p-value = 0.010). Split analysis by age group has revealed that reduced height velocity was significantly higher in females than males of the adolescent’s group (90.5% compared to 62.5%, respectively, Chi-square = 4.194, p-value = 0.040), and not in the children group. Reduced height velocity was not associated with any of the other factors (Table 4).

**Table 4:** Comparing height velocity measured in two consecutive years by other parameters

	*N*	Normal in both years		Delayed in One/two years		Chi-square	*p*-value
		*n*	%	*n*	%		
Age group							
2–9	25	6	24.0	19	76.0	0.048	0.826
10–18	37	8	21.6	29	78.4		
Gender							
Male	30	11	36.7	19	63.3	6.597	***0.010***
Female	32	3	9.4	29	90.6		
Nationality							
UAE	30	9	30.0	21	70.0	3.487	0.175
Arabs	9	3	33.3	6	66.7		
Non-Arabs	21	2	9.5	19	90.5		
DFO (Deferoxamine) Chelation Drug							
No	50	12	24.0	38	76.0	0.298	0.717
Yes	12	2	16.7	10	83.3		
DFP (Deferiprone) Chelation Drug							
No	59	13	22.0	46	78.0	0.209	0.543
Yes	3	1	33.3	2	66.7		
DFX (Deferasirox) Chelation Drug							
No	15	3	20.0	12	80.0	0.075	1.000
Yes	47	11	23.4	36	76.6		

The prevalence of pre-diabetes did not differ significantly by age group, gender, nationality, BMI, or type of chelation drug (Table 5). Prediabetic patients (*n* = 12) had a median Ferritin level of 2466 (IQR: 2091–3539). The prevalence of hypothyroidism among children and adolescents, was significantly higher among non-Arabs (26.9%) compared to UAE nationals (4.5%) and Arabs (4.2%; Chi-square = 10.028, p-value = 0.007). Among all study participants, the prevalence of subclinical hypothyroidism was 9.5% (*n* = 10), and none had primary hypothyroidism.

**Table 5:** Prevalence of pre-diabetes by type of drug chelation & other parameters

	*N*	FBG < 100		100 ≤ FBG < 126		Chi-square	*p*-value
		*n*	%	*n*	%		
Age group							
2–9	2	2	100.0	0	0.0	0.696	1.000
10–18	46	34	73.9	12	26.1		
Gender							
Male	24	18	75.0	6	25.0	0.000	1.000
Female	24	18	75.0	6	25.0		
Nationality							
UAE	24	19	79.2	5	20.8	0.895	0.639
Arabs	9	6	66.7	3	33.3		
Non-Arabs	12	8	66.7	4	33.3		
BMI							
< 18.5	17	14	82.4	3	17.6	0.932	0.628
18.5–24.9	26	18	69.2	8	30.8		
>=25.0	4	3	75.0	1	25.0		
DFO (Deferoxamine) Chelation Drug							
No	41	31	75.6	10	24.4	0.056	1.000
Yes	7	5	71.4	2	28.6		
DFP (Deferiprone) Chelation Drug							
No	46	34	73.9	12	26.1	0.696	1.000
Yes	2	2	100.0	0	0.0		
DFX (Deferasirox) Chelation Drug							
No	6	5	83.3	1	16.7	0.254	1.000
Yes	42	31	73.8	11	26.2		

## Discussion

Endocrine complications is common among patients with BTM, of which short stature seems the most prevalent (49%) [20]. Males were found to have higher prevalence than females in prior studies, however, our study found females to have statistically significant higher percentage of reduced height velocity in females. There are several factors contributing to such a high rate including iron overload particularly in the liver iron concentration of > 15 mg Fe/g dry weight [21]. Furthermore, pituitary iron overload has been postulated as an etiology for growth failure with malfunction of somatotrophs, in addition to defective hepatic growth hormones receptors [22, 23]. Studies have reported high prevalence of growth hormone deficiency [20]. Menarche occurred at a later age ( 13.5 years) in girls with BTM in comparison with the mean age for menarche in the general population in UAE which is 12.6 years [24]. This delay in menarche could partly explain why females are more affected than males in terms of reduced growth velocity.

Due to the small number of patients with Hb less than 9 g/dL, it is hard to conclude the association between low Hb and reduced height velocity among children and adolescents with BTM in our study. However, other studies have reported significant difference in pretransfusion hemoglobin and ferritin level between children with normal height and those with stunted growth [25].

Glucose dysregulation has been well documented in patients with BTM due to different etiologies including iron deposition in the beta cells in the pancreas which occur typically after the first decade of life, zinc deficiency, and hepatitis C [26–28]. More than a quarter (26.5%) of adolescents treated at our centre were found to be in a prediabetes state. This is comparable to other published data from different regions [29, 30]. Although relative insulin deficiency is the most common cause for the pre-diabetic state, obesity and its associated insulin resistance is known to accelerate the onset of diabetes. Therefore, it is important that clinicians advise healthy lifestyle including healthy diet and regular physical activity to delay the onset of diabetes.

Thyroid gland seem to be affected in patients with BTM and the impact of hypothyroidism shows mostly after the age of 10. There is a wide range of prevalence of hypothyroidism ranging 13–60% in different parts of the world [9, 31–37]. We found 9.1% prevalence of subclinical hypothyroidism and none with primary hypothyroidism suggesting that this might occur after adolescent years.

BMT is the preferred choice of treatment when matched donor is available [38–41]. Although many endocrinological complications seem to occur despite bone marrow transplant, gonadal insufficiency is found to be significantly lower among children who undergone transplant early in life [42].

## Conclusion

Despite the effective treatment protocols in the UAE, growth delay remains the main concern for this group of patients. effective treatment is key to further reducing endocrinopathies. Although the sample size is limited, we postulate that the low percentage of endocrinopathies among children with BTM treated at Dubai thalassemia center and the low level of pretransfusion anemia reflect the effective transfusion and chelation at the center.

## Data Availability

The datasets used and/or analysed during the current study available from the corresponding author on reasonable request.
